# Anterior Versus Posterior Surgical Approaches in Degenerative Cervical Myelopathy: A Single-Center Retrospective Comparative Study of 102 Patients

**DOI:** 10.3390/jcm15145494

**Published:** 2026-07-13

**Authors:** Leonardo Anselmi, Michele Da Broi, Aria Nouri, Nasser Bouhalassa, Gianpaolo Jannelli, Alexandre Lavé, Fabio Bouras, Abiram Sandralegar, Granit Molliqaj, Federico Pessina, Enrico Tessitore

**Affiliations:** 1 Department of Neurosurgery, IRCCS Humanitas Research Hospital, Via Manzoni 56, Rozzano, 20089 Milan, Italy; 2 Division of Neurosurgery, Department of Clinical Neurosciences, Faculty of Medicine, Geneva University Hospitals and University of Geneva, 4 Rue Gabrielle-Perret-Gentil, 1205 Geneva, Switzerland; micheledabroi92@gmail.com (M.D.B.); aria.nouri@hug.ch (A.N.); nasser.bouhalassa@hug.ch (N.B.); gianpaolo.jannelli@hug.ch (G.J.); alexandre.lave@hug.ch (A.L.); fabio.bouras@hug.ch (F.B.); abiram.sandralegar@hug.ch (A.S.); granit.molliqaj@hug.ch (G.M.); enrico.tessitore@hug.ch (E.T.); 3 Department of Neurosurgery, Neurocenter of South Switzerland, Ente Ospedaliero Cantonale, 6500 Lugano, Switzerland; 4 Department of Biomedical Sciences, Humanitas University, Via Rita Levi Montalcini 4, Pieve Emanuele, 20072 Milan, Italy

**Keywords:** degenerative cervical myelopathy, anterior cervical surgery, posterior cervical decompression, surgical outcomes, postoperative complications, surgical approach selection

## Abstract

**Background/Objectives:** Degenerative cervical myelopathy (DCM) is the leading cause of non-traumatic spinal cord dysfunction in adults. Both anterior and posterior surgical approaches are widely used for its treatment, yet their comparative impact on clinical outcomes and complication profiles remains clinically relevant. This study aimed to describe early and one-year postoperative outcomes between anterior and posterior surgical strategies in a consecutive single-center cohort. **Methods**: We conducted a retrospective single-center study of 102 consecutive adult patients surgically treated for DCM between 2013 and 2024, with complete datasets and a minimum one-year follow-up. Surgical approaches were classified as anterior, posterior, or combined. Clinical outcomes were assessed using the modified Japanese Orthopaedic Association scale (mJOA), Neck Disability Index (NDI), and Numeric Rating Scale for arm pain (NRS-arm) preoperatively, at 4–6 weeks and at one year. Complications were systematically recorded and stratified by approach. Nonparametric tests were used for all comparisons (significance threshold *p* < 0.05). **Results**: Anterior procedures were performed in 82 patients (80.4%), posterior in 18 (17.6%), and combined in 2 (2.0%). No significant between-group differences were observed in neurological, functional, or pain outcomes at either time point (ΔmJOA *p* = 0.268; ΔNDI *p* = 0.632; ΔNRS *p* = 0.562 at one year). Complications occurred in 13 patients (12.7%), with approach-specific profiles: anterior surgery was associated with hematoma, dysphagia, and dysphonia, posterior surgery with CSF leak, wound infection, and kyphosis. No C5 palsy was recorded. **Conclusions**: Both anterior and posterior surgical approaches were followed by neurological and functional improvement at one year. Given the descriptive nature of the study and the baseline differences between groups, these findings should not be read as a formal comparison of effectiveness, but they reinforce the importance of individualized, pathology-driven surgical planning in DCM.

## 1. Introduction

Degenerative cervical myelopathy (DCM) is the most common cause of non-traumatic spinal cord dysfunction in adults and represents a major cause of progressive neurological disability worldwide [1,2]. With the aging of the population and the increasing prevalence of degenerative spinal conditions, the clinical burden of DCM is expected to rise further in the coming decades [2,3]. Once myelopathy becomes clinically manifest, surgical decompression remains the only effective treatment capable of halting disease progression and enabling neurological recovery [2,4].

DCM arises from a combination of static and dynamic spinal cord compression related to degenerative changes such as disc degeneration, osteophyte formation, hypertrophy of the ligamentum flavum, and ossification of the posterior longitudinal ligament, often in association with altered sagittal alignment and segmental instability [3,5]. Prolonged spinal cord compression leads to secondary injury mechanisms including ischemia, neuroinflammation, demyelination, and axonal degeneration, which account for the progressive and potentially irreversible course of the disease if left untreated [5,6].

Several surgical strategies are currently employed for the treatment of DCM. Anterior approaches allow direct decompression of ventral pathology and correction of focal kyphosis, whereas posterior approaches provide indirect multilevel decompression through posterior cord drift and are frequently selected in the presence of extensive stenosis or multilevel disease with preserved cervical lordosis [7,8]. Both strategies are well established and widely used, with approach selection primarily driven by anatomical, biomechanical, and disease-related factors rather than by expectations of differential clinical efficacy.

Although both anterior and posterior decompression have been associated with favorable clinical outcomes in patients with DCM, differences in surgical indications, disease extent, and outcome measures across studies have resulted in a broad range of reported outcomes. In addition, anterior approaches have been more frequently associated with complications such as postoperative dysphagia, recurrent laryngeal nerve palsy, and approach-related visceral injuries, whereas posterior decompression has been more commonly linked to C5 nerve root palsy, wound-related complications, increased blood loss, and longer hospital stay [4,9]. In this context, comparative analyses derived from consecutive institutional cohorts with standardized outcome assessment may provide complementary insights into postoperative recovery patterns following different surgical strategies [4].

The aim of the present study was therefore to compare early and one-year clinical outcomes following anterior versus posterior surgical approaches in a single-center consecutive cohort of patients treated for degenerative cervical myelopathy. Using standardized neurological, functional, and pain-related outcome measures, we aimed to provide additional real-world evidence on postoperative recovery patterns and to contribute original data to the ongoing discussion on surgical approach selection in DCM.

## 2. Materials and Methods

### 2.1. Study Design and Patient Population

The present study is a retrospective secondary analysis of data prospectively collected within a study approved by the Ethics Committee of Geneva University Hospitals (protocol 11-194; NAC 11-067). Consecutive patients surgically treated for degenerative cervical myelopathy (DCM) between 2013 and 2024 were included. Inclusion criteria comprised: (1) adult patients (≥18 years) with a clinical diagnosis of DCM, defined as objective signs of spinal cord dysfunction including upper motor neuron signs, gait disturbance, hand dysfunction, or sphincter involvement, in the absence of an alternative neurological diagnosis; (2) radiological confirmation of cervical spinal cord compression on MRI, with or without intramedullary T2 signal changes; (3) surgical decompression performed via an anterior, posterior, or combined approach; and (4) complete preoperative and one-year postoperative clinical outcome data. Exclusion criteria included isolated cervical radiculopathy without myelopathic signs, non-degenerative etiologies (trauma, infection, tumor), previous cervical spine surgery, and incomplete follow-up at one year.

### 2.2. Radiological Assessment

Preoperative cervical MRI and lateral radiographs were analyzed for all included patients. Cervical sagittal alignment was assessed on lateral radiographs using the C2–C7 Cobb angle and classified as lordotic (>40°), normal (10–40°), or kyphotic (≤10°). Compression morphology was classified on MRI as anterior, when cord compression originated exclusively from ventral structures, posterior, when compression originated exclusively from dorsal structures, or circumferential, when both anterior and posterior compression elements were identified.

Intramedullary signal changes were assessed on T1- and T2-weighted sagittal MRI sequences and classified as: T2 hyperintensity only, reflecting cord edema or gliosis, or combined T1 hypointensity and T2 hyperintensity, interpreted as indicative of more advanced myelomalacic transformation. Patients without intramedullary signal change on MRI were nonetheless included in the analysis, as they presented with clinically manifest myelopathy and clear radiological evidence of cord compression.

### 2.3. Surgical Strategy Classification

Surgical management was categorized according to surgical approaches and procedure types. Approaches were classified as anterior, posterior or combined anterior–posterior. Within the anterior group, procedures included anterior cervical discectomy and fusion (ACDF) and anterior cervical corpectomy and fusion (ACCF). Within the posterior group, procedures included posterior decompression alone and posterior decompression combined with posterior fixation. The choice of surgical strategy was based on anatomical, biomechanical, and disease-related considerations, including the extent and location of compression, number of involved levels, and sagittal alignment.

### 2.4. Clinical Outcome Measures

Clinical outcomes were assessed using standardized neurological, functional, and pain-related scales, including the modified Japanese Orthopaedic Association (mJOA) score (17-point scale according to Keller) [10], the Neck Disability Index (NDI), and the Numeric Rating Scale for arm pain (NRS-arm). Outcomes were evaluated preoperatively, at early postoperative follow-up (4–6 weeks), and at one-year follow-up when available. Changes in clinical status were expressed as Δ values calculated as follow-up minus baseline values.

### 2.5. Complications

Postoperative complications were retrospectively recorded for the purpose of this analysis. Complications were classified as overall and approach-related, and included neurological complications (e.g., new or worsened neurological deficit, C5 nerve root palsy), wound-related complications (including surgical site infection), approach-specific complications (such as dysphagia or dysphonia following anterior surgery, considered postoperative complications only when persisting beyond 2 weeks, a temporal threshold based on the expected resolution of soft-tissue oedema after anterior cervical exposure), and other perioperative adverse events.

### 2.6. Statistical Analysis

Statistical analyses were performed using jamovi statistical software (The jamovi project, version 2.7.14.0). Continuous variables were reported as median and interquartile range (IQR) and compared between groups using the Mann–Whitney U test or Kruskal–Wallis test, as appropriate. Categorical variables were expressed as counts and percentages and compared using the chi-square test or Fisher’s exact test when expected cell counts were <5. All tests were two-tailed, and a *p* value <0.05 was considered statistically significant.

## 3. Results

### 3.1. Patient Population and Surgical Distribution

Between January 2013 and December 2024, 219 patients surgically treated for degenerative cervical myelopathy were screened. After exclusions, 102 patients with complete clinical and radiological datasets and at least one year of follow-up were included in the final analysis. Of the 117 excluded patients, 70 were excluded due to absence of informed consent and 47 due to incomplete follow-up, most commonly related to postoperative dropout or missed visits.

The mean age at the time of surgery was 57.6 ± 11.0 years (range 36–80), with a balanced sex distribution (51.0% males, 49.0% females).

Compression morphology and sagittal alignment differed between surgical groups. Anterior cord compression was present in 64 patients (62.7%) and circumferential compression in 38 patients (37.3%); no patient presented with isolated posterior compression. Circumferential compression was more frequent in the posterior group (83.3%) than in the anterior group (26.8%). Kyphotic alignment was the most frequent pattern overall, observed in 50.6% of anterior patients and 37.5% of posterior patients; both combined patients presented with kyphotic alignment.

Regarding intramedullary signal changes, T2 hyperintensity was present in 81 patients (79.4%), while combined T1 and T2 signal changes were observed in 4 patients (3.9%). Seventeen patients (16.7%) showed no intramedullary signal change on preoperative MRI.

The anterior approach represented the most frequently adopted surgical strategy and was performed in 82 patients (80.4%), followed by posterior-only procedures in 18 patients (17.6%) and combined anterior–posterior surgery in 2 patients (2.0%).

With regard to specific surgical techniques, anterior cervical discectomy and fusion (ACDF) was performed in 69 cases (67.6%), while anterior cervical corpectomy and fusion (ACCF) accounted for 9 cases (8.8%). Posterior decompression alone was used in 4 patients (3.9%), whereas posterior decompression combined with fixation was performed in 14 cases (13.7%). In addition, 6 patients (5.9%) underwent multi-technique procedures involving multiple techniques during the index surgery. (Table 1 and Table 2).

### 3.2. Comparative Analysis of Surgical Approaches

**Figure 1:**
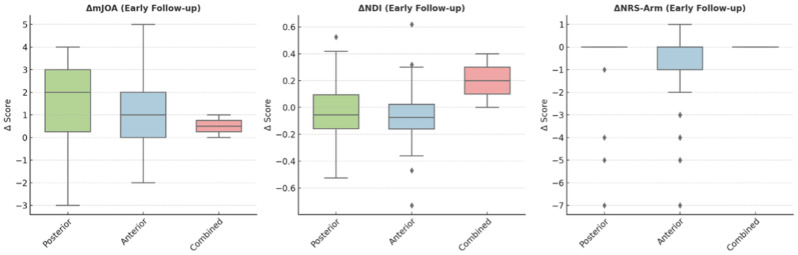
Boxplot of early postoperative outcome changes (ΔmJOA, ΔNDI, ΔNRS-arm) by surgical approach (anterior, posterior, combined).

At the early postoperative follow-up (4–6 weeks), no significant differences were observed between anterior, posterior, and combined approaches across the three outcome measures. The median neurological improvement (ΔmJOA) was slightly higher in the posterior group compared to anterior and combined (2.0 vs 1.0 and 0.5, respectively), although this did not reach statistical significance (χ^2^ = 2.51, *p* = 0.285). The functional disability improvement (ΔNDI) and radicular pain reduction (ΔNRS) were also comparable across groups, with Kruskal–Wallis tests showing non-significant results (ΔNDI χ^2^ = 2.73, *p* = 0.256; ΔNRS χ^2^ = 0.64, *p* = 0.725) (Table 3 and Figure 1).

Results involving the combined anterior–posterior group (*n* = 2) should be interpreted with extreme caution given the very limited sample size, which substantially reduces the reliability of any statistical comparison involving this group.

**Figure 2:**
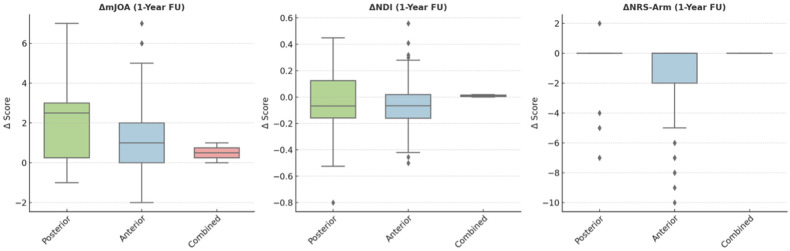
Boxplot of one-year postoperative outcome changes (ΔmJOA, ΔNDI, ΔNRS-arm) by surgical approach (anterior, posterior, combined).

At one-year follow-up, neurological improvement appeared greater in the posterior group (median ΔmJOA 2.5) compared with anterior (median 1.0) and combined approaches (median 0.5); however, this difference did not reach statistical significance (χ^2^ = 2.63, *p* = 0.268). Functional outcomes (ΔNDI) and radicular pain reduction (ΔNRS-arm) remained comparable across approaches, with no statistically significant differences (ΔNDI χ^2^ = 0.92, *p* = 0.632; ΔNRS χ^2^ = 1.15, *p* = 0.562) (Table 4 and Figure 2).

### 3.3. Comparative Analysis of Surgical Procedures

**Figure 3:**
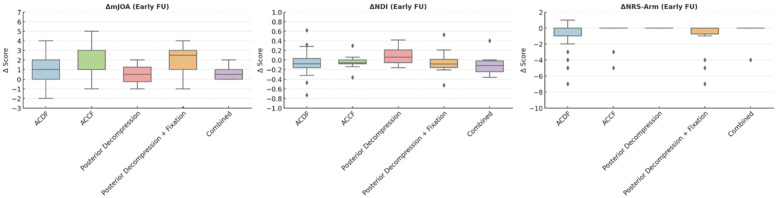
Boxplot of early postoperative outcome changes (ΔmJOA, ΔNDI, ΔNRS-arm) by surgical procedure.

At the early follow-up, neurological improvement (ΔmJOA) was slightly higher in patients treated with posterior decompression with fixation (median 2.5) compared to ACDF (median 1.0), ACCF (median 1.0), posterior decompression alone (median 0.5), and multi-technique procedures (median 0.5). However, these differences did not reach statistical significance (χ^2^ = 6.60, *p* = 0.159). Functional improvement (ΔNDI) and pain reduction (ΔNRS) were also comparable across procedures, with Kruskal–Wallis tests confirming the absence of significant differences (ΔNDI χ^2^ = 2.33, *p* = 0.675; ΔNRS χ^2^ = 1.60, *p* = 0.808) (Table 5 and Figure 3).

**Figure 4:**
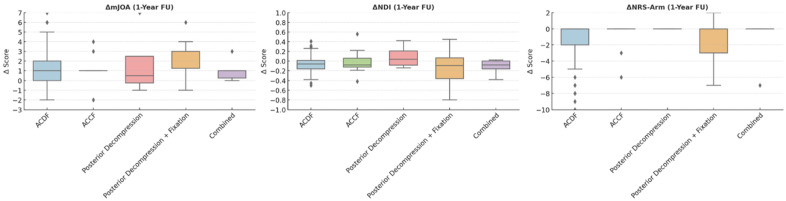
Boxplot of one-year postoperative outcome changes (ΔmJOA, ΔNDI, ΔNRS-arm) by surgical procedure.

At the 1-year follow-up, neurological improvement (ΔmJOA) was greatest after posterior decompression with fixation (median 3.0) versus ACDF (median 1.0), ACCF (median 1.0), posterior decompression alone (median 0.5), and multi-technique procedures (median 1.0). Differences did not reach statistical significance (χ^2^ = 3.95, *p* = 0.412). Functional improvement (ΔNDI) and radicular pain reduction (ΔNRS) were comparable across procedures (ΔNDI χ^2^ = 1.95, *p* = 0.745; ΔNRS χ^2^ = 1.73, *p* = 0.785) (Table 6 and Figure 4).

### 3.4. Postoperative Complications

Postoperative complications occurred in 13 patients (12.7%). The most frequent event was postoperative hematoma (*n* = 3; 2.9%). Surgical site infection, dysphagia, and dysphonia each occurred in 2 patients (2.0%), while cerebrospinal fluid (CSF) leak, Horner’s syndrome, postoperative kyphosis, and implant-related failure were observed in 1 patient each (1.0%). No cases of C5 nerve root palsy were recorded. (Table 7)

Postoperative complications were analysed across the three surgical groups (anterior, posterior, combined) in the cohort with complete 1-year follow-up (*n* = 102).

In the anterior cohort (*n* = 82), the most frequent events were hematoma (3.7%), dysphagia (2.4%), and dysphonia (2.4%). Isolated cases of infection (1.2%), Horner’s syndrome (1.2%), and implant-related complication (1.2%) were also observed. No cases of CSF leak or C5 palsy were recorded.

In the posterior cohort (*n* = 18), cerebrospinal fluid leak (5.6%), infection (5.6%), and kyphosis (5.6%) occurred in one case each. No cases of dysphagia, dysphonia, implant-related complications, or C5 palsy were recorded.

**Figure 5:**
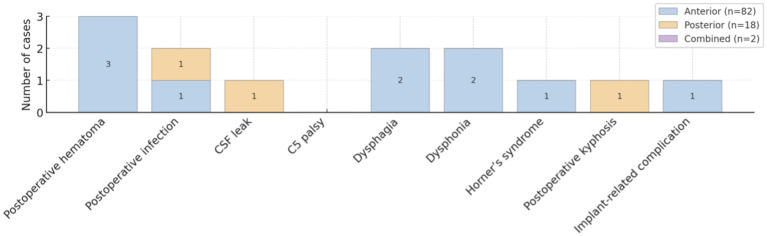
Bar chart of postoperative complications by surgical approach.

No complications were documented in the combined anterior- posterior group (*n* = 2) (Table 8 and Figure 5).

**Figure 6:**
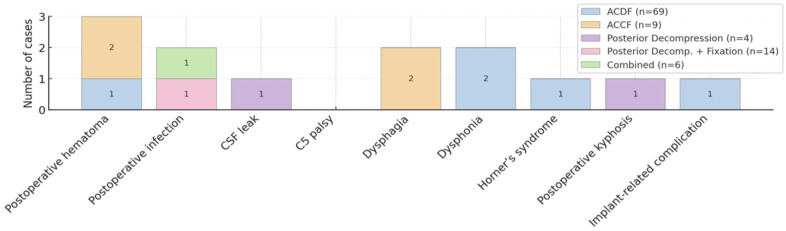
Bar chart of postoperative complications by surgical procedure.

When postoperative complications were analyzed across the five surgical procedures, the following patterns were observed, none of which reached statistical significance with Fisher’s exact test. Postoperative hematoma was numerically most frequent after ACCF (22.2%; *p* = 0.379). Infection showed a higher numerical frequency in the multi-technique procedures group (16.7%; *p* = 0.358). CSF leak occurred exclusively after posterior decompression (25.0%; *p* = 0.246). Dysphagia was numerically most frequent following ACCF (22.2%; *p* = 0.325). Postoperative kyphosis occurred exclusively after posterior decompression (25.0%; *p* = 0.246). Dysphonia (*p* = 1.000), Horner’s syndrome (*p* = 1.000), and implant-related complications (*p* = 1.000) did not show notable differences across procedures (Table 9 and Figure 6).

Wilcoxon rank-sum tests were conducted to evaluate whether postoperative complications influenced 1-year functional outcomes, measured as changes in neurological status (ΔmJOA), disability (ΔNDI), and pain (ΔNRS).

When functional outcomes at 1 year were compared between patients with and without complications, no statistically significant differences were found in most cases.

**Figure 7:**
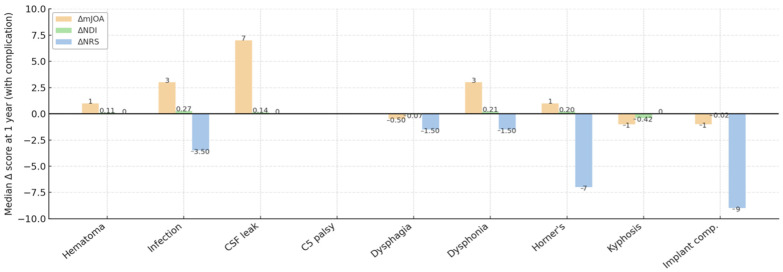
Bar chart of one-year outcome changes (ΔmJOA, ΔNDI, ΔNRS-arm) in patients with postoperative complications.

A non-significant trend toward worse radicular pain improvement was observed in patients with implant-related complications (ΔNRS, *p* = 0.093), while no corresponding differences were detected in neurological recovery as assessed by mJOA (Table 10 and Figure 7).

## 4. Discussion

Surgical decompression represents the cornerstone of treatment for degenerative cervical myelopathy, and both anterior and posterior approaches are widely accepted and routinely employed in clinical practice. Anterior strategies are typically preferred for focal ventral compression and kyphotic alignment, whereas posterior approaches are preferentially selected in cases of multilevel stenosis, dorsal pathology, or preserved cervical lordosis. Despite these well-established indications, the relative impact of surgical approach on postoperative neurological recovery and functional outcomes remains a matter of ongoing debate.

In the present series, both anterior and posterior approaches were associated with clinically meaningful neurological and functional improvement at early and one-year follow-up. Although numerically greater gains were observed after posterior strategies, no statistically significant differences emerged between approaches. Given the substantial baseline differences between groups and the absence of statistical adjustment, these observations remain descriptive. They are nonetheless in line with the broader concept that adequate spinal cord decompression, rather than the surgical corridor itself, is a key determinant of neurological recovery in DCM. It should be noted, however, that the absence of statistically significant differences does not constitute evidence of equivalence between approaches, given the limited sample size and group imbalance of the present study.

This observation is consistent with the prospective AOSpine North America and International studies, which demonstrated substantial neurological improvement following both approaches with mean mJOA gains of approximately 2–3 points at one year [11,12], and with subsequent pooled analyses confirming comparable functional outcomes when baseline severity is accounted for [13,14,15].

The numerically higher median mJOA improvement observed after posterior approaches in our cohort should therefore be interpreted with caution. Patients selected for posterior surgery typically presented with more extensive multilevel disease and greater baseline neurological impairment, a factor known to influence the magnitude of postoperative recovery through a floor–ceiling effect. Accordingly, the greater absolute improvement observed after posterior procedures likely reflects differences in baseline severity and patient selection rather than an intrinsic superiority of posterior surgery [16,17].

Beyond the comparison between anterior and posterior surgical corridors, further insight can be gained by examining outcomes according to the specific surgical procedures employed. When outcomes were further stratified by procedure type, posterior decompression with fixation achieved the highest median neurological improvement at one year. Again, this finding did not reach statistical significance and is best interpreted in the context of surgical indication. Posterior fixation-based strategies were preferentially employed in patients with advanced multilevel stenosis, instability, or sagittal imbalance—factors known to influence both baseline disability and postoperative recovery trajectories [18]. Functional outcomes and radicular pain relief did not differ significantly across procedures, in line with the concept that adequacy of decompression and appropriate patient selection are key determinants of outcome.

The lack of statistically significant differences in functional outcomes between strategies in the present series is consistent with the broader body of contemporary evidence. Large systematic reviews and meta-analyses have consistently shown that neurological recovery after surgery for DCM is more strongly influenced by baseline disease severity, symptom duration, and patient-related factors than by the chosen surgical approach. In this context, Tetreault et al. found no meaningful differences in postoperative neurological improvement between anterior and posterior procedures, while Sattari et al. similarly demonstrated comparable functional and neurological gains between ACDF and posterior decompression for multilevel disease when baseline characteristics were accounted for in the analysis [9,13]. These converging data are consistent with current guidelines favoring individualized, pathology-driven surgical planning over a prescriptive preference for either approach [2,19].

While neurological and functional outcomes did not differ significantly across surgical strategies, postoperative complications revealed distinct and predictable approach-specific patterns. The overall complication rate in our cohort (12.7%) was slightly lower than those reported in large prospective AOSpine series, where rates between 15% and 25% have been described [11,12]. This difference may reflect careful patient selection, standardized perioperative management, and the predominance of ACDF procedures in our population.

Anterior procedures were primarily associated with approach-related complications, including postoperative hematoma, dysphagia, dysphonia, Horner’s syndrome, and implant-related events, with no cases of cerebrospinal fluid leak or postoperative kyphosis. These findings are consistent with the established risk profile of anterior cervical surgery, particularly with respect to soft tissue dissection and esophageal retraction time [9,20]. Within the anterior group, corpectomy was associated with the highest numerical rates of hematoma (22.2%) and dysphagia (22.2%), corroborating prior meta-analytic evidence demonstrating increased perioperative morbidity for corpectomy compared with multilevel discectomy [20], although these differences did not reach statistical significance in our cohort.

Posterior approaches, although performed in a smaller subset of patients, showed a numerically higher proportion of structural complications, cerebrospinal fluid leakage, postoperative infection, and kyphotic deformity [9]. The exclusive occurrence of postoperative kyphosis after posterior decompression without fixation in our cohort, though not statistically significant given the limited sample size, is consistent with classical and contemporary literature describing post-laminectomy instability and deformity occurring particularly in the absence of supplemental stabilization [21,22,23]. Importantly, increasing evidence indicates that postoperative alignment and sagittal balance play a central role in long-term spinal stability following cervical decompression. In particular, preoperative sagittal malalignment and inadequate restoration or preservation of cervical lordosis have been associated with a higher risk of mechanical complications and delayed deterioration, especially after posterior decompression performed without fixation [24,25]. Accordingly, the indication for posterior fixation should be carefully tailored to the individual preoperative alignment and balance.

No cases of C5 palsy were observed, although its absence should not be interpreted as evidence of reduced risk given the limited sample size [26,27].

From a functional standpoint, postoperative complications did not significantly affect one-year neurological or disability outcomes in our cohort. This finding is consistent with prior studies suggesting that, while complications may prolong recovery or necessitate reintervention, long-term neurological outcomes often remain preserved following adequate decompression [28]. A non-significant trend toward poorer radicular pain improvement was observed in patients with implant-related complications. Although underpowered, this observation is clinically plausible, as hardware-related issues may preferentially affect radicular symptoms without necessarily compromising central neurological recovery.

Overall, these descriptive findings are consistent with the broader literature reporting favorable neurological and functional outcomes after both anterior and posterior approaches in appropriately selected patients [9,15]. Differences between strategies lie predominantly in their complication profiles, which are largely predictable and procedure specific [15]. These observations are concordant with contemporary AO Spine clinical practice recommendations emphasizing tailored surgical decision-making to optimize both neurological recovery and long-term spinal stability [19].

### Limitations

Several limitations inherent to the retrospective single-center design must be acknowledged. Surgical approach selection was driven by anatomical and disease-related factors, introducing confounding by indication that could not be fully adjusted for given the sample size. Furthermore, as baseline characteristics could not be compared between included and excluded patients, the magnitude and direction of potential selection bias remain unknown. The distribution of approaches was uneven, reflecting real-world surgical practice rather than randomized allocation, and the combined group was too small for meaningful comparison. Given these constraints, the findings of this study are best interpreted as descriptive and hypothesis-generating, complementing rather than replacing prospective comparative evidence. The absence of statistically significant differences between approaches should be interpreted cautiously in this context. Preoperative radiological characterization, including sagittal alignment and compression morphology, was incorporated as baseline descriptive data but not as covariates in the primary comparative outcome analysis. K-line status, cervical sagittal vertical axis, and T1 slope were not systematically recorded, and these omissions should be considered when interpreting approach-related decision-making. Follow-up was limited to one year, which may not capture late complications or adjacent segment disease progression.

## 5. Conclusions

Both anterior and posterior surgical approaches showed meaningful neurological and functional improvement at one year. Given the descriptive nature of the study and the baseline differences between groups, these findings should not be read as a formal comparison of effectiveness, but they reinforce the importance of individualized, pathology-driven surgical planning in DCM. Differences between strategies were primarily reflected in predictable, approach-specific complication profiles. Prospective studies with larger and more balanced cohorts will be needed to further characterize the comparative outcomes of anterior and posterior approaches in DCM.

## Figures and Tables

**Table 1:** Distribution of surgical approaches and procedures in the study cohort (*n* = 102).

	Variable	*n*	%
**Surgical approach**	Anterior	82	80.4
	Posterior-only	18	17.6
	Combined anterior–posterior approach	2	2.0
**Surgical procedure**	ACDF	69	67.6
	ACCF	9	8.8
	Posterior decompression	4	3.9
	Posterior decompression + fixation	14	13.7
	Multi-technique procedures	6	5.9

**Table 2:** Baseline demographic and clinical characteristics stratified by surgical approach.

	Variable	Total (*n* = 102)	Anterior (*n* = 82)	Posterior (*n* = 18)	Combined (*n* = 2)
**Demographics**	Age, years—median (IQR)	58 (49–66)	55 (47–64)	63 (59–74)	64 (59–69)
	Male sex—n (%)	52 (51.0%)	40 (48.8%)	10 (55.6%)	2 (100.0%)
**Preoperative clinical scores**	mJOA—median (IQR)	14 (12–15)	14 (12–16)	12 (11–14)	15 (15–15)
	NDI—median (IQR)	0.29 (0.16–0.46)	0.29 (0.16–0.46)	0.39 (0.17–0.54)	0.05 (0–0.10)
	NRS-arm—median (IQR)	0 (0–3)	0 (0–3)	0 (0–0)	0 (0–0)
**Myelopathy severity (mJOA)**	Mild (mJOA 15–17)—*n* (%)	43 (42.2%)	38 (46.3%)	3 (16.7%)	2 (100.0%)
	Moderate (mJOA 12–14)—*n* (%)	35 (34.3%)	27 (32.9%)	8 (44.4%)	0 (0.0%)
	Severe (mJOA ≤ 11)—*n* (%)	24 (23.5%)	17 (20.7%)	7 (38.9%)	0 (0.0%)
**Disease extent**	Single-level disease—*n* (%)	31 (30.4%)	31 (37.8%)	0 (0.0%)	0 (0.0%)
	Multi-level disease—*n* (%)	71 (69.6%)	51 (62.2%)	18 (100.0%)	2 (100.0%)
	Operated levels—median (IQR)	2 (1–3)	2 (1–2)	3 (3–4)	4 (2–6)
**Radiological characteristics**	Anterior cord compression—*n* (%)	64 (62.7%)	60 (73.2%)	3 (16.7%)	1 (50.0%)
	Circumferential compression—*n* (%)	38 (37.3%)	22 (26.8%)	15 (83.3%)	1 (50.0%)
	Lordotic C2–C7 (>40°)—*n* (%) ^†^	2 (2.1%)	2 (2.5%)	0 (0.0%)	0 (0.0%)
	Normal C2–C7 (10–40°)—*n* (%) ^†^	47 (48.5%)	37 (46.8%)	10 (62.5%)	0 (0.0%)
	Kyphotic C2–C7 (≤10°)—*n* (%) ^†^	48 (49.5%)	40 (50.6%)	6 (37.5%)	2 (100.0%)
**MRI signal changes**	T2 signal only—*n* (%)	81 (79.4%)	61 (74.4%)	18 (100.0%)	2 (100.0%)
	T1 + T2 signal—*n* (%)	4 (3.9%)	4 (4.9%)	0 (0.0%)	0 (0.0%)
	No signal change—*n* (%) ^‡^	17 (16.7%)	17 (20.7%)	0 (0.0%)	0 (0.0%)

^†^ Sagittal alignment data were not available for 5 patients (3 anterior, 2 posterior); percentages calculated on available data (*n* = 97: anterior *n* = 79, posterior *n* = 16). ^‡^ Patients without intramedullary signal change presented with clinically manifest myelopathy and radiological cord compression in the absence of T2 hyperintensity.

**Table 3:** Comparative analysis of clinical outcome changes (ΔmJOA, ΔNDI, ΔNRS-arm) by surgical approach at early postoperative follow-up (4–6 weeks).

Outcome	Approach	Mean (±sd)	Median	(*n*)	Kruskal–Wallis χ^2^ (df = 2)	*p*-Value
**ΔmJOA**	Anterior	1.05 ± 1.40	1.0	82	2.51	0.285
	Posterior	1.50 ± 1.92	2.0	18		
	Combined	0.50 ± 0.71	0.5	2		
**ΔNDI**	Anterior	−0.06 ± 0.18	−0.075	82	2.73	0.256
	Posterior	−0.02 ± 0.24	−0.056	18		
	Combined	0.20 ± 0.28	0.20	2		
**ΔNRS**	Anterior	−0.91 ± 1.82	0.0	82	0.64	0.725
	Posterior	−0.94 ± 2.10	0.0	18		
	Combined	0.00 ± 0.00	0.0	2		

**Table 4:** Comparative analysis of clinical outcome changes (ΔmJOA, ΔNDI, ΔNRS-arm) by surgical approach at one-year follow-up.

Outcome	Approach	Mean (±sd)	Median	(*n*)	Kruskal–Wallis χ^2^ (df = 2)	*p*-Value
**ΔmJOA**	Anterior	1.43 ± 1.83	1.0	82	2.63	0.268
	Posterior	2.17 ± 2.31	2.5	18		
	Combined	0.50 ± 0.71	0.5	2		
**ΔNDI**	Anterior	−0.06 ± 0.19	−0.065	82	0.92	0.632
	Posterior	−0.08 ± 0.32	−0.068	18		
	Combined	0.01 ± 0.01	0.01	2		
**ΔNRS**	Anterior	−1.40 ± 2.60	0.0	82	1.15	0.562
	Posterior	−1.17 ± 2.64	0.0	18		
	Combined	0.00 ± 0.00	0.0	2		

**Table 5:** Comparative analysis of clinical outcome changes (ΔmJOA, ΔNDI, ΔNRS-arm) by surgical procedure at early postoperative follow-up (4–6 weeks).

Outcome	Procedure	Mean (±sd)	Median	(*n*)	Kruskal–Wallis χ^2^ (df = 4)	*p*-Value
**ΔmJOA**	ACDF	0.99 ± 1.37	1.0	69	6.60	0.159
	ACCF	1.67 ± 1.80	1.0	9		
	Posterior Decompression	0.50 ± 1.29	0.5	4		
	Posterior Decompression + Fixation	1.79 ± 2.01	2.5	14		
	Multi-technique procedures	0.67 ± 0.82	0.5	6		
**ΔNDI**	ACDF	−0.06 ± 0.19	−0.080	69	2.33	0.675
	ACCF	−0.04 ± 0.17	−0.060	9		
	Posterior Decompression	0.09 ± 0.25	0.059	4		
	Posterior Decompression + Fixation	−0.05 ± 0.24	−0.084	14		
	Multi-technique procedures	−0.08 ± 0.27	−0.115	6		
**ΔNRS**	ACDF	−0.91 ± 1.84	0.0	69	1.60	0.808
	ACCF	−0.89 ± 1.83	0.0	9		
	Posterior Decompression	0.00 ± 0.00	0.0	4		
	Posterior Decompression + Fixation	−1.21 ± 2.33	0.0	14		
	Multi-technique procedures	−0.67 ± 1.63	0.0	6		

**Table 6:** Comparative analysis of clinical outcome changes (ΔmJOA, ΔNDI, ΔNRS-arm) by surgical procedure at one-year follow-up.

Outcome	Procedure	Mean (±sd)	Median	(*n*)	Kruskal–Wallis χ^2^ (df = 4)	*p*-Value
**ΔmJOA**	ACDF	1.46 ± 1.89	1.0	69	3.95	0.412
	ACCF	1.22 ± 1.64	1.0	9		
	Posterior Decompression	1.75 ± 3.59	0.5	4		
	Posterior Decompression + Fixation	2.29 ± 1.98	3.0	14		
	Multi-technique procedures	1.00 ± 1.10	1.0	6		
**ΔNDI**	ACDF	−0.06 ± 0.17	−0.060	69	1.95	0.745
	ACCF	−0.00 ± 0.28	−0.080	9		
	Posterior Decompression	0.09 ± 0.25	0.036	4		
	Posterior Decompression + Fixation	−0.13 ± 0.33	−0.095	14		
	Multi-technique procedures	−0.11 ± 0.15	−0.080	6		
**ΔNRS**	ACDF	−1.43 ± 2.63	0.0	69	1.73	0.785
	ACCF	−1.00 ± 2.12	0.0	9		
	Posterior Decompression	0.00 ± 0.00	0.0	4		
	Posterior Decompression + Fixation	−1.50 ± 2.93	0.0	14		
	Multi-technique procedures	−1.17 ± 2.86	0.0	6		

**Table 7:** Postoperative complications in the overall cohort (*n* = 102).

Variable	*n*	%
Postoperative hematoma	3	2.94
Infection	2	1.96
Dysphagia	2	1.96
Dysphonia	2	1.96
CSF leak	1	0.98
Horner’s syndrome	1	0.98
Kyphosis	1	0.98
Implant-related failure	1	0.98
C5 palsy	0	0.0

**Table 8:** Postoperative complications stratified by surgical approach.

Approach	Hematoma	Infection	CSF Leak	C5 Palsy	Dysphagia	Dysphonia	Horner’s	Kyphosis	Implant Complication
**Anterior (*n* = 82)**	3 (3.7%)	1 (1.2%)	0 (0.0%)	0 (0.0%)	2 (2.4%)	2 (2.4%)	1 (1.2%)	0 (0.0%)	1 (1.2%)
**Posterior (*n* = 18)**	0 (0.0%)	1 (5.6%)	1 (5.6%)	0 (0.0%)	0 (0.0%)	0 (0.0%)	0 (0.0%)	1 (5.6%)	0 (0.0%)
**Combined (*n* = 2)**	0 (0.0%)	0 (0.0%)	0 (0.0%)	0 (0.0%)	0 (0.0%)	0 (0.0%)	0 (0.0%)	0 (0.0%)	0 (0.0%)

**Table 9:** Postoperative complications stratified by surgical procedure.

Approach	Hematoma	Infection	CSF Leak	C5 Palsy	Dysphagia	Dysphonia	Horner’s	Kyphosis	Implant Complication
**ACDF (*n* = 69)**	1 (1.4%)	0 (0.0%)	0 (0.0%)	0 (0.0%)	0 (0.0%)	2 (2.9%)	1 (1.4%)	0 (0.0%)	1 (1.4%)
**ACCF (*n* = 9)**	2 (22.2%)	0 (0.0%)	0 (0.0%)	0 (0.0%)	2 (22.2%)	0 (0.0%)	0 (0.0%)	0 (0.0%)	0 (0.0%)
**Posterior** **Decompression (*n* = 4)**	0 (0.0%)	0 (0.0%)	1 (25.0%)	0 (0.0%)	0 (0.0%)	0 (0.0%)	0 (0.0%)	1 (25.0%)	0 (0.0%)
**Posterior** **Decomp. +** **Fixation (*n* = 14)**	0 (0.0%)	1 (7.1%)	0 (0.0%)	0 (0.0%)	0 (0.0%)	0 (0.0%)	0 (0.0%)	0 (0.0%)	0 (0.0%)
**Multi-** **technique** **procedures (*n* = 6)**	0 (0.0%)	1 (16.7%)	0 (0.0%)	0 (0.0%)	0 (0.0%)	0 (0.0%)	0 (0.0%)	0 (0.0%)	0 (0.0%)

**Table 10:** Wilcoxon rank-sum test *p*-values for the association between individual postoperative complications and one-year clinical outcome changes (ΔmJOA, ΔNDI, ΔNRS-arm).

Complication	*n*	ΔMJOA (*p*)	ΔNDI (*p*)	ΔNRS (*p*)
Postoperative hematoma	3	0.1911	0.9921	0.9921
Infection	2	0.1409	0.0935	0.4471
CSF leak	1	0.0895	0.5410	0.6588
C5 palsy	0	–	–	–
Dysphagia	2	0.1652	0.3718	0.6728
Dysphonia	2	0.3783	0.1195	0.6728
Horner’s syndrome	1	0.7728	0.2215	0.1307
Postoperative kyphosis	1	0.1489	0.0995	0.6588
Implant-related complication	1	0.1489	0.4347	0.0927

## Data Availability

All relevant data are fully reported within the study. Additional information can be requested from the corresponding authors.

## Abbreviations

The following abbreviations are used in this manuscript:

ACDF	Anterior Cervical Discectomy and Fusion
ACCF	Anterior Cervical Corpectomy and Fusion
CSF	Cerebrospinal Fluid
DCM	Degenerative Cervical Myelopathy
IQR	Interquartile Range
mJOA	Modified Japanese Orthopaedic Association score
NDI	Neck Disability Index
NRS	Numeric Rating Scale
NRS-arm	Numeric Rating Scale for Arm Pain
SD	Standard Deviation
